# Morphological and molecular analysis of rose cultivars from the Grandiflora and Kordesii garden groups

**DOI:** 10.18699/vjgb-24-07

**Published:** 2024-02

**Authors:** S.S. Yudanova, O.V. Dorogina, O.Yu. Vasilyeva

**Affiliations:** Central Siberian Botanical Garden of the Siberian Branch of the Russian Academy of Sciences, Novosibirsk, Russia; Central Siberian Botanical Garden of the Siberian Branch of the Russian Academy of Sciences, Novosibirsk, Russia; Central Siberian Botanical Garden of the Siberian Branch of the Russian Academy of Sciences, Novosibirsk, Russia

**Keywords:** Rosa L., grandiflora, Rosa Kordesii, ISSR markers, morphological characters, phenological observations, Rosa L., грандифлора, розы Кордеса, ISSR-маркеры, морфологические признаки, фенологиче-ские наблюдения

## Abstract

The breeding of remontant rose cultivars that are resistant to diseases and adverse conditions, with high decorative value and continuous flowering is the most important task during work with the gene pool of garden roses. Currently, intercultivar hybridization within a single garden group has largely outlived its usefulness. It is necessary to breed for highly decorative forms or cultivars that have outstanding resistance, morphological characters and patterns of seasonal rhythms, and use these plants as parental forms in further breeding. This study represents a comparative analysis of rose cultivars from two garden groups, Grandiflora (Gurzuf, Lezginka, Korallovy Syurpriz, Queen Elizabeth, Komsomolsky Ogonyok, Love) and Rosa Kordesii (Letniye Zvyozdy, Dortmund, Gutsulochka). These cultivars proved themselves during many years of testing in harsh climatic conditions. The objectives of the study were to determine the genetic relationship within the groups and to assign phenotypically different cultivars to one or another garden group. The analysis was carried out by morphological, phenological and ISSR markers. According to the phenological observations on the Grandiflora cultivars, Komsomolsky Ogonyok had later budding and flowering stages. Polymorphic data generated from the ISSR markers showed that this cultivar was the most distant from the others and formed a separate cluster on the dendrogram. A comparison of the morphological characters (flower diameter, number of petals, peduncle length, bush height) showed a significant difference ( p < 0.05) between Komsomolsky Ogonyok and the other Grandiflora cultivars. A dendrogram based on a molecular analysis showed a lack of close relationships between Komsomolsky Ogonyok and the Kordesii group, which formed a separate cluster. A pairwise comparison of the morphological characters in Komsomolsky Ogonyok with the Kordesii group revealed a significant ( p <0.05) difference in three of the four characters studied. The exceptions were flower diameter when comparing with Dortmund and Letniye Zvyozdy and peduncle length when comparing with Gutsulochka. Although Komsomolsky Ogonyok has a pattern of seasonal development similar to Dortmund in the Kordesii group, the molecular analysis did not assign the former to this group of roses. The cultivars that have valuable characters that no average rose does and that are phenotypically different from such roses represent the most valuable breeding material.

## Abstract (ru)

Поиск зимостойких, устойчивых к грибным болезням сортов, характеризующихся высокой декоративностью, ремонтантным и продолжительным цветением, является важнейшей задачей при работе с коллекционным генофондом садовых роз. В настоящее время межсортовая гибридизация роз в пределах одной садовой группы во многом исчерпала себя. Требуется поиск высокодекоративных форм или сортов, выделяющихся по резистентности, морфологическим и ритмологическим признакам для использования в селекции в качестве родительских форм. В работе выполнен сравнительный анализ сортов из двух садовых групп – грандифлора (Гурзуф, Лезгинка, Коралловый сюрприз, Queen Elizabeth, Комсомольский огонёк, Love) и кордезии (Летние звёзды, Dortmund, Гуцулочка). Эти сорта хорошо показали себя в течение многих лет испытаний в суровых климатических условиях. Целью исследования было определение степени родства внутри групп и установление возможной принадлежности фенотипически различающихся сортов к одной из групп. Анализ проводили по морфологическим, фенологическим признакам, а также с помощью ISSR-маркеров. По результатам фенологических наблюдений в группе грандифлора выделился сорт Комсомольский огонёк: более позднее вступление в фазы бутонизации и цветения. Данные полиморфизма, полученные на основании ISSR-маркирования, пока- зали, что он удален от других сортов, образуя на дендрограмме отдельный кластер. Сравнение сортов роз по морфологическим признакам (диаметр цветка, количество лепестков, длина цветоноса, высота куста) также свидетельствует о достоверных отличиях ( p <0.05) сорта Комсомольский огонёк от остальных сортов группы грандифлора. Дендрограмма, построенная на основании молекулярного анализа, показала отсутствие близ- кого родства сорта Комсомольский огонёк и группы кордезии, которые формировали отдельный кластер. При попарном сравнении морфологических показателей сорта Комсомольский огонёк и группы кордезии обнару- жено достоверное различие ( p < 0.05) по трем из четырех изученных признаков, за исключением диаметра цветка (при сравнении с сортами Dortmund и Летние звёзды) и длине цветоноса (при сравнении с Гуцулочкой). Несмотря на то что Комсомольский огонёк по феноритмике схож с сортом Dortmund из группы кордезии, моле- кулярный анализ не позволяет отнести его к данной группе роз. Такие сорта, фенотипически отличающиеся от общей массы и обладающие рядом ценных признаков, являются ценнейшим селекционным материалом

## Introduction

Roses (Rosa L.) are among the oldest plants cultivated by man not only for decorative use but also for perfumery, medical and culinary purposes. The genus includes about 200 species; however, only 10–15 of them have contributed to the garden groups of modern roses (Cairns, 2007). According to the modern classification, the world’s entire collection represented by 40,000 cultivars is subdivided into 36 horticultural groups (Annotated Catalog ..., 2018; Plugatar et al., 2019). The most valuable quality of the cultivars from the Tea-Hybrid, Floribunda, Grandiflora, Rosa Kordesii, Polyanthus and Miniature groups is their ability for remontant flowering (Klimenko, 2010; Gorodnyaya, 2014; Tyshchenko, 2015), which is biologically conditioned by the presence in their hereditary basis of the genetic material from the evergreen species of section Indicae that do not tolerate winter well and are not prone to winter dormancy.

One of the main criteria for selecting garden rose cultivars promising for cultivation in harsh climatic conditions is flowering of their annual shoots (Vasilyeva, 1999). However, this biological feature is not characteristic of all garden groups and not of all species of the same garden group, e. g. most cultivars of large-flowered climbing (LCl.) roses produce generative shoots on perennial shoot formation systems (SFSs) that die almost every year due to severe winters (Pashina, 2011; Kapelyan, 2017; Plugatar et al., 2018). For that reason, in terms of rose gardens grown in continental climate, along with the Tea-Hybrid and Floribunda, such groups as Grandiflora and Rosa Kordesii are of great interest.

The Grandiflora (Gr.) roses were bred in the 1950s solely based on their morphological characters and with no regard to the origin, they resulted from crossed Floribunda and Tea- Hybrid roses. They are praised for their abundant and remontant flowering, as that of the Floribunda, as well as for the long straight shoots with large flowers of different colors resembling those of the Tea-Hybrid. Unlike the latter, the Grandiflora roses grow not single flowers but small-flower inflorescences. The most important character of the group has been their growth strength and higher winter hardiness if compared to Tea-Hybrid roses

Kordes Roses, or Rosa Kordesii, is a relatively young garden group, selected from a spontaneous Rosa rugosa × Rosa wichuraiana hybrid by the W. Kordes’ Sohne Company, whose breeding priority has been selection of unpretentious and winter-hardy forms. Their features are abundant flowering from June to late fall, high winter hardiness and increased resistance to diseases (Bardakova, 2017; Adritskaya, Kapelyan, 2022). In harsh climates, these roses grow flowers on annual shoots, which can make them a substitute for the climbing roses flowering on perennial SFSs and poorly surviving Siberian winters.

A striking representative of Rosa Kordesii is the Dortmund roses that are often used in breeding as a parental form for being resistant to fungal diseases. In the Nikitsky Botanical Garden, Z.K. Klimenko cultivated such Rosa Kordesii cultivars as Letniye Zvyozdy and Gutsulochka (Klimenko, Rubtsova, 1986) that have proved to be highly decorative and stable in harsh climatic conditions. Queen Elizabeth is the most popular representative of the Grandiflora group, known for its decorative features and complex resistance, and for these reasons it has been repeatedly used in breeding. Among the Russian members of the group, Komsomolsky Ogonyok (Charlotte Wheatcroft × Gloria Dei cross) is the most popular. Its long-term trials carried out in the Central Siberian Botanical Garden of the Siberian Branch of the Russian Academy of Sciences (CSBG SB RAS) demonstrated that it was phenotypically different from the Grandiflora group but had similarities with Rosa Kordesii. For that reason, the research presented in this paper was to evaluate the cultivars from the Grandiflora and Rosa Kordesii groups based on their morphological and molecular genetic characters in order to determine the kinship within the groups and to possibly establish whether the phenotypically distinguished cultivars belonged to one of the groups mentioned

An additional goal was investigating CSBG SB RAS collection’s gene pool for valuable forms to perform further breeding. Considering Siberia’s harsh continental climate, we searched for cultivars of high winter hardiness, resistant to fungal diseases and characterized by remontant and prolonged flowering.

To evaluate the collection’s genetic polymorphism, ISSR (inter simple sequence repeats) analysis was employed. The technique interprets the DNA sequences flanked by microsatellite loci, has good reproducibility and does not require cloning and sequencing of DNA fragments for primer selection, thereby significantly reducing its cost and labor intensity Considering that the number of microsatellite repeats is very high in the genome in both animals and plants, this method is a very convenient tool for genetic analysis (Amom, Nongdam, 2017; Dorogina, Zhmud, 2020).

## Materials and methods

In our study, we investigated the roses of the Grandiflora (Gurzuf, Lezginka, Korallovy Syurpriz, Queen Elizabeth, Komsomolsky Ogonyok, Love) and Rosa Kordesii (Letniye Zvyozdy, Dortmund, Gutsulochka) groups. The Grandiflora roses had (1) small inflorescences of large flowers in different colors; (2) tall bushes reaching up to 2 m in height if grown in southern Russia; (3) large and glossy leaves; (4) abundant, remontant flowering; (5) sufficiently high winter hardiness, which is favorable for Siberia. The Kordesii roses, on the other hand, were characterized by high winter hardiness, resistance to diseases and abundant long flowering. In a continental climate, this group can partially substitute climbing roses because they flower on annual shoots

The roses’ morphobiological characters such as flower diameter, number of petals, peduncle length, and bush height were studied during five summer seasons from 2017 to 2021. The study was carried out at CSBG SB RAS’s Collections of Living Plants in Open and Protected Grounds, USU 440534 (54°49ʹ13.8ʺ N 83°06ʹ13.3ʺ E). To evaluate the morphobiological characters, standard methods were applied (Methodology of State Variety Testing…, 1968; Klimenko et al., 2019; Suprun, 2021). Phenological observations were carried out according to I.N. Beideman’s method (1974) with modifications (Fomina, 2012). Student’s t-test was used to confirm the reliability of the differences obtained for metric characters (Haynes, 2013). Average mean and standard error М ± x were calculated using 20 plants.

For DNA isolation, the СTAB method with some modifications was used (Doyle J.J., Doyle J.L., 1987). Amplification was carried out according to the following program: primary denaturation for 2 min at 95 °С; 35 amplification cycles – denaturation for 20 sec at 94 °С, primer annealing for 45 sec, elongation for 1.5 min at 72 °С; final elongation for 7 min at 72 °С. PCR and further electrophoretic separation of amplification products were performed in 1–1.5 % agarose gel in 1× TBE buffer according to standard methods (Vasilyeva et al., 2020). The list of ISSR primers used in this work, their characteristics and annealing temperatures are given in Table 1.

**Table 1.**
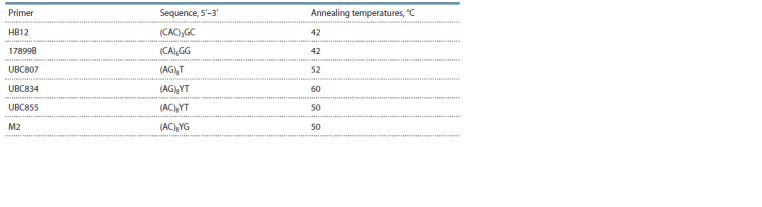
Characteristics of ISSR primers used to study Grandiflora and Kordes Roses

Quantitative assessment of marker polymorphism and determination of the level of divergence between the studied forms were performed in a binary matrix where the presence or absence of PCR fragments of equal size was denoted as 1 or 0. For statistical data processing, the TREECON software (Van de Peer, Wacher, 1994) was used. The genetic distances were calculated as:

**Formula 1.**
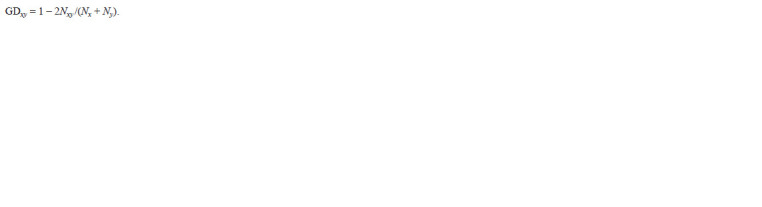
Formula 1

Here, Nxy is the number of total fragments for samples x and y, Nx and Ny are the number of fragments for samples x and y, respectively (Nei, Li, 1979).

The nearest neighbors algorithm with bootstrap support of at least 100 was employed to build ISSR marker distribution dendrograms. The polymorphism level (P, %) of each primer was calculated by the formula:

**Formula 2.**
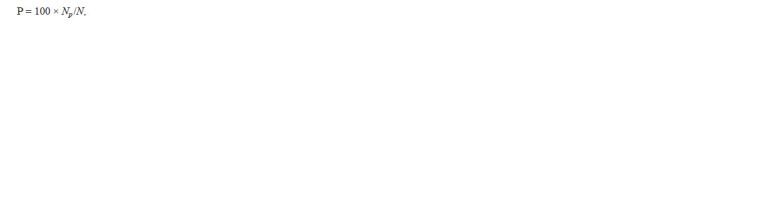
Formula 2

where Np is the number of polymorphic fragments and N is the total number of fragments

## Results

The vegetation period of roses in Siberia includes the following stages: aftergrowth; current-year shooting; budding; first (I) and second (II) flowering; defloration. As for more favorable climatic conditions in some, mainly southern, regions of Russia, garden roses have a third (III) flowering stage. The longtime average annual data accumulated during phenological observations of 2017–2022 demonstrated that the Grandiflora roses had earlier shooting regrowth than the Kordesii ones, despite the winter shelter being removed from the entire collection at the same time (Table 2). The time required for the shoots to produce the first flowers is more extended in the Kordesii roses, which is due to the more powerful shoots with a greater number of internodes than those of the Grandiflora; however, reflowering was less prolonged in the Kordesii roses.

**Table 2.**
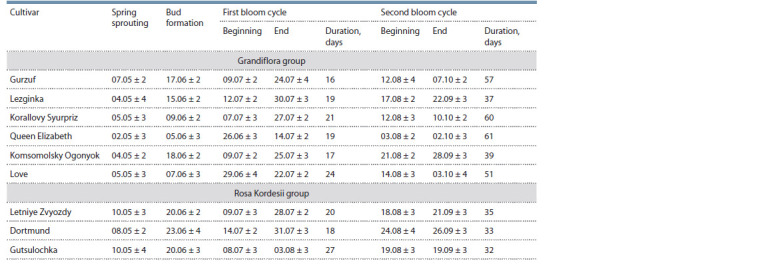
Long-term phenological data of rose cultivars from the Grandiflora and Kordes Roses groups (Novosibirsk, 2017–2021)

Among the studied cultivars, the earliest flowering was observed in the foreign Grandiflora roses (Love and Queen Elizabeth). The time Queen Elizabeth entered the flowering phase on average came at the 61st day after removing winter shelters and beginning of vegetation, which was significantly ( p < 0.01) different from all the cultivars studied, except for Love: its time to flowering was close to that of Queen Elizabeth. The other cultivars bloomed in 72–79 days from the beginning of vegetation with Lezginka and Komsomolsky Ogonyok being the latest-blooming in the Grandiflora group

Study of the phenological phases demonstrated that Komsomolsky Ogonyok entered the budding phase later than all the other grandiflora flowers, so Komsomolsky Ogonyok, by this indicator, was closer to the Kordesii. Moreover, Komsomolsky Ogonyok’s second flowering started later than that of other representatives of the Grandiflora group and was similar to that of the Kordesii roses. Its phenorhythmics1 turned out to be closest to that of the Dortmund cultivar from the Kordesii group (see Table 2).Phenorhythmics describes the phenological rhythms of growth and development of organisms, adapted to the seasonal rhythm of environmental factors and expressed in a clear alternation of phenological phases. The alternation of phenophases is illustrated by phenospectra (Dedu, 1989).

Such morphological characters as flower diameter, number of petals, peduncle length and rose-bush height were investigated. The cultivars were compared separately by garden groups according to the classification of the World Federation of Rose Societies (WFRS). The analysis demonstrated that Komsomolsky Ogonyok also significantly differed from the other cultivars (Table 3): it had statistically significant differences ( p <0.05) for all characters with Queen Elizabeth and Lezginka, as well as for three characters (flower diameter, number of petals and bush height) with Korallovy Syurpriz. Comparison against Gurzuf and Love also showed statistically significant differences ( p < 0.05) for two characters (flower diameter, number of petals). The found pheno- and morphological characters gave us grounds to assume that Komsomolsky Ogonyok should not be referred to the Grandiflora group because its small flower diameter and small number of petals made it closer to the Kordesii roses

**Table 3.**
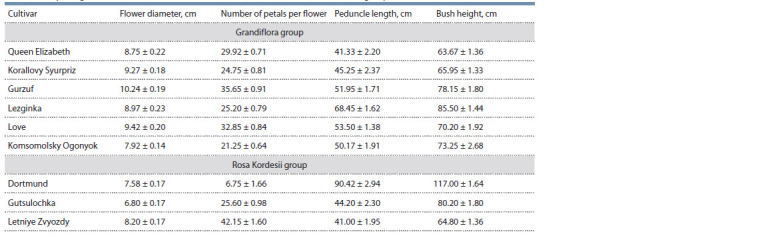
Morphological characters of rose cultivars from the Grandiflora and Kordes Roses groups

However, comparing Komsomolsky Ogonyok’s morphometric characters to those of the Kordesii revealed statistically significant differences as well ( p <0.05): it differed from Dortmund and Letniye Zvyozdy by the number of petals, flower stalk and bush heights, and from Gutsulochka by flower diameter, number of petals and bush height

So, the analyzed phenological phases and morphometric characters showed that Komsomolsky Ogonyok differed from the cultivars of both groups. To assess the degree of kinship, ISSR analysis was employed

**Fig. 1.**
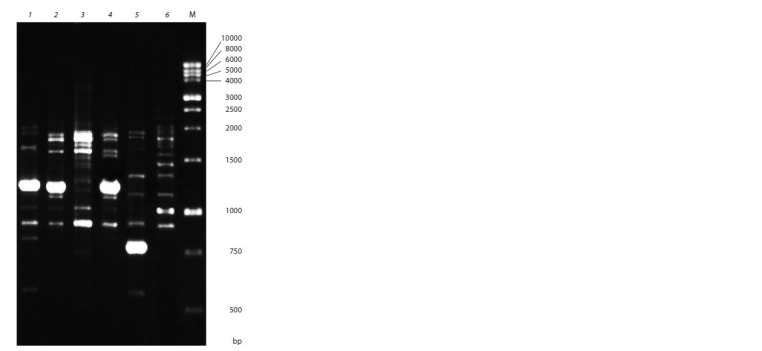
Electrophoregram of PCR products obtained by DNA amplification with ISSR primer HB12 – (CAC)3GC. Tracks with designation of samples: 1 – Komsomolsky Ogonyok, 2 – Queen Elizabeth, 3 – Korallovy Syurpriz, 4 – Lezginka, 5 – Gurzuf, 6 – Love; track M – DNA marker.

DNA amplification with six ISSR primers identified 122 PCR fragments ranging from 250 to 3,000 bp in length, including 109 polymorphic ones. The number of amplification fragments ranged from 18 (markers HB12 and UBC834) to 23 (17899B) (Fig. 1). The level of polymorphism detected by a single primer ranged from 77.8 % (HB12) to 94.4 % (UBC855) and averaged to 91.42 %.

**Fig. 2.**
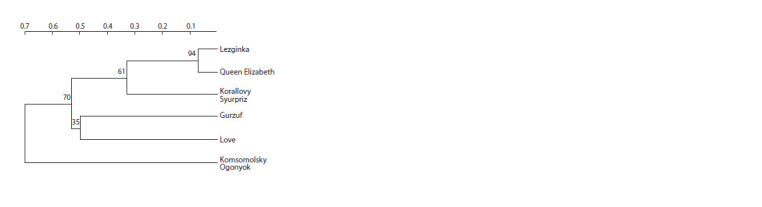
Dendrogram built with the Neighbor-Joining algorithm based on PCR spectra data of cultivars from the Grandiflora group Numbers at nodes show the level of statistical support (100 bootstrap replicates). Numbers at the top show genetic distance.

Based on the obtained results, the samples in the study were divided into three clusters (Fig. 2): Cluster I including Lezginka, Queen Elizabeth and Korallovy Syurpriz; Cluster II including Gurzuf and Love. Komsomolsky Ogonyok was found to be the most distant from the other cultivars and formed a separate Cluster III.

Queen Elizabeth, bred in the middle of the 20th century, has been widely used for breeding new cultivars, and most likely was a parental one for the Lezginka roses bred in the Nikitsky Botanical Garden in 2005, which was evidenced by the statistically significant2 (>90) genetic distance (0.1).Confidence measure for a node of interest of >70 (95 % CI) is considered as highly reliable.

**Fig. 3.**
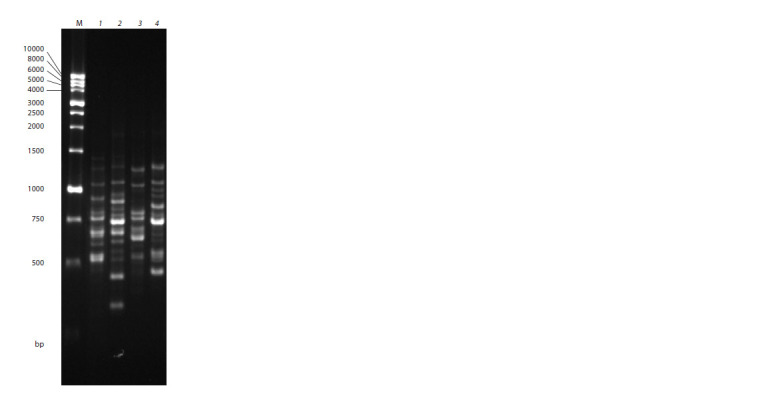
Electrophoregram of PCR products obtained by DNA amplification with ISSR primer UBC855 – (AC)8YT. Tracks with designation of samples: 1 – Dortmund, 2 – Letniye Zvyozdy, 3 – Komsomolsky Ogonyok, 4 – Gutsulochka; track M – DNA marker.

At the next stage of our study, we compared Komsomolsky Ogonyok with the cultivars from the Kordesii group. This comparison revealed 103 amplified fragments ranging from 350 to 2,000 bp in length, including 97 polymorphic ones. The total number of identified fragments ranged from 15 (UBC855) to 19 (HB12) (Fig. 3). The level of polymorphism detected by a single primer ranged from 88.9 % (17899B) to 100 % (M2) and averaged to 94.25 %.

**Fig. 4.**
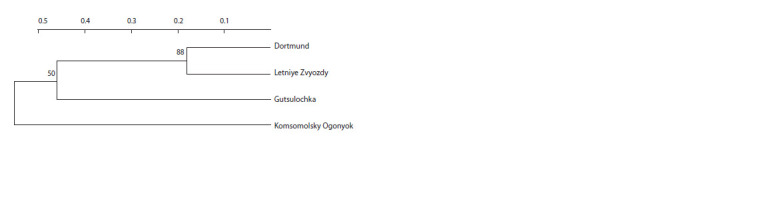
Dendrogram built based on ISSR-PCR data for cultivars from the Kordes Roses group. Numbers at nodes show the level of statistical support (100 bootstrap replicates). Numbers at the top show genetic distance

The comparison showed no affinity between the Komsomolsky Ogonyok and Kordesii roses (Fig. 4). The highest kinship score was found for Dortmund and Letniye Zvyozdy. Apparently, the Dortmund cultivar was a parental one in this pair. Gutsulochka was also found to be related to the Dortmund and Letniye Zvyozdy cultivars, but their kinship was less pronounced and its statistical significance was somewhat lower, so these three cultivars of the Kordesii group were brought into Cluster IV.

Thus, the results of molecular analysis as well as investigation of the pheno- and morphometric parameters of the roses in the two groups have shown that Komsomolsky Ogonyok stands out from the members of both groups.

## Discussion

The classification system for garden roses has been refined and undergone various, sometimes diametrically opposed, changes over the past 50 years; until the 1970s, the world’s garden roses amounted to approximately 25,000 cultivars subdivided into 30 garden groups (Bylov et al., 1972). In the 1980s (Klimenko, Rubtsova, 1986), foreign specialists in rose breeding and varietal evaluation reduced the number of garden groups to 16. One of the most prominent examples of this merger was the Rambler (climbing roses) group now uniting the Multiflora and Wichuraiana roses. Initially, these groups had clear differences, as they were bred from two different species belonging to the same Synstylae section, Rosa multiflora Thunb. and R. wichuraiana, respectively. But further crosses between these groups resulted in cultivars with the characters common to the original species, and obvious distinction between the groups disappeared. Remarkably, modern molecular genetic studies (Cui et al., 2020) still distinct the original species.

The genetic studies to clarify taxonomic, phylogenetic relationships in roses are intensively developing. They include building genetic, genomic and transcriptomic tools to investigate the molecular mechanisms underlying the creation of some rose species (Bendahmane et al., 2013; Duta-Cornescu et al., 2017; Li et al., 2018). In particular, the genetic kinship of the Taif roses to some rose genotypes (Rosa sp.) was assessed based on random amplified polymorphic DNA and simple sequence repeat markers inter and simple (El-Assal et al., 2014), and Chinese researchers identified the transcripts common to the rose family, which should help clarify the phylogenetic relationships in it (Li et al., 2018).

Rose introduction and breeding has had a long and complex history since the plant has been crossbred in completely different regions of the world, such as Europe, Asia and the Middle East. Domesticating the rose, breeders have concentrated on several characters affecting flower quality such as periodic flowering; terry flowers; petal coloration and fragrance (Bendahmane et al., 2013). So, stimulation of flowering, flower longevity, and creation of novelty in flower structure, color range and fragrances are the main objectives of ornamental plant breeding today. New genome editing techniques offer new opportunities to study the rose’s gene function and develop new cultivars for the floriculture industry (Giovannini et al., 2021). Knowing the genetic structure of a species, genus or family allows for more rational use of the available gene pool by optimizing initial breeding forms selection

Over the past two decades, the molecular basis of floral fragrance as well as its genetic inheritance have been studied in the rose, providing useful information for both researchers and manufacturers (Yan et al., 2014; Shi, Zhang, 2022). A complex research carried out by French, Chinese and German scientists has led to the sequencing of the genome of the tea rose (Rosa chinensis), a progenitor species for many modern cultivars, and the genes presumably responsible for its remontancy have been discovered. They have also found that the synthesis of the volatiles giving the flower its fragrance and the pigments responsible for its color are coordinated by the same tandem of a protein and a non-coding microRNA (Raymond et al., 2018).

## Conclusion

As has been mentioned above, the world’s entire assortment of garden and park roses exceeds 40,000 cultivars subdivided into 36 garden groups. Many of them have no clear confirmation of their origin, e. g., the only indication of the Dortmund cultivar’s origin in the catalogs is Seedling × R. kordesii. In this situation, creating scientific collections of roses, their gene pools, and passportization of their cultivars becomes of great importance, because intervarietal rose hybridization within the same garden group has largely exhausted itself. It is necessary to search for new cultivars, primarily among the Tea-Hybrid, Floribunda and Grandiflora groups. Apart from high ornamentality, these cultivars are to have distinguishing morphological, rhythmological and resistance characters for use in breeding as paternal and maternal forms.

In the present study, rose cultivars from the bioresource scientific collection of CSBG SB RAS (USU 440534) belonging to the Grandiflora and Kordesii garden groups were investigated. The study has demonstrated that the Komsomolsky Ogonyok cultivar has different molecular genetic, pheno- and morphological characters than those of the Grandiflora group it belongs to. At the same time, it is not closely related to the investigated Kordesii cultivars, despite being close to them in flower size and phenorhythmics.

Such cultivars that are phenotypically different from others and possess a number of valuable characters – primarily winter hardiness, resistance to fungal diseases, and decorative, remontant and prolonged flowering – are valuable breeding material. Our research has shown it is Komsomolsky Ogonyok that can be recommended for breeding rose cultivars for regions with harsh climatic conditions. The polymorphism revealed from ISSR marking data can be used for molecular genetic passportization, which is a necessary step for accounting and conservation of the gene pool of valuable cultivars. The advantage of this approach is that the ISSR technique is polylocus, has a large number of PCR amplification products and does not require sequencing in polyacrylamide gels.

## Conflict of interest

The authors declare no conflict of interest.
